# Increased risk of nephrolithiasis: an emerging issue in children with congenital adrenal hyperplasia due to 21-hydroxylase deficiency

**DOI:** 10.1007/s12020-024-03792-6

**Published:** 2024-03-27

**Authors:** Mariangela Chiarito, Crescenza Lattanzio, Vito D’Ascanio, Donatella Capalbo, Paolo Cavarzere, Anna Grandone, Francesca Aiello, Giorgia Pepe, Malgorzata Wasniewska, Thomas Zoller, Mariacarolina Salerno, Maria Felicia Faienza

**Affiliations:** 1 Pediatric Unit, Department of Precision and Regenerative Medicine and Ionian Area, University of Bari “A. Moro”, Bari, Italy; 2 Institute of Sciences of Food Production (ISPA), Italian National Research Council (CNR), Bari, Italy; 3 Pediatric Endocrinology Unit-Department of Translational Medical Sciences, University of Naples Federico II and University Hospital Federico II, Endo-ERN Center for Rare Endocrine Conditions, Naples, Italy; 4 Pediatric Division, Department of Pediatrics, University Hospital of Verona, Verona, Italy; 5 Department of Woman, Child and General and Specialized Surgery, University of Campania Luigi Vanvitelli, Naples, Italy; 6 Department of Human Pathology of adulthood and childhood, University of Messina, Messina, Italy

**Keywords:** Congenital adrenal hyperplasia (CAH), 21-hydroxylase deficiency (21-OHD), Nephrolithiasis, ACTH, 17-OHP

## Abstract

**Purpose:**

To investigate the incidence of nephrolithiasis in a cohort of children with congenital adrenal hyperplasia (CAH), and to study if there is an association with the metabolic control of the disease.

**Methods:**

This study was designed as a multicenter 1 year-prospective study involving 52 subjects (35 males) with confirmed molecular diagnosis of CAH due to 21-hydroxylase deficiency (21-OHD). Each patient was evaluated at three different time-points: T0, T1 (+6 months of follow-up), T2 (+12 months of follow up). At each follow up visit, auxological data were collected, and adrenocorticotrophic hormone (ACTH), 17-hydroxyprogesterone (17-OHP), Δ4-androstenedione, dehydroepiandrosterone sulfate (DHEAS) serum levels, and urinary excretion of creatinine, calcium, oxalate and citrate were assayed. Moreover, a renal ultrasound was performed.

**Results:**

The incidence of nephrolithiasis, assessed by ultrasound was 17.3% at T0, 13.5% at T1 and 11.5% at T2. At T0, one subject showed nephrocalcinosis. In the study population, a statistically significant difference was found for 17-OHP [T0: 11.1 (3.0–25.1) ng/mL; T1: 7.1 (1.8–19.9) ng/mL; T2: 5.9 (2.0–20.0) ng/mL, *p* < 0.005], and Δ4-androstenedione [T0: 0.9 (0.3–2.5) ng/mL; T1: 0.3 (0.3–1.1) ng/mL; T2: 0.5 (0.3–1.5) ng/mL, *p* < 0.005] which both decreased over the follow up time. No statistically significant difference among metabolic markers was found in the group of the subjects with nephrolithiasis, even if 17-OHP, DHEAS and Δ4-androstenedione levels showed a tendency towards a reduction from T0 to T2. Principal component analysis (PCA) was performed to study possible hidden patterns of associations/correlations between variables, and to assess the trend of them during the time. PCA revealed a decrease in the amount of the variables 17-OHP, Δ4-androstenedione, and ACTH that occurred during follow-up, which was also observed in subjects showing nephrolithiasis.

**Conclusions:**

our data demonstrated that children affected with 21-OHD can be at risk of developing nephrolithiasis. Additional studies are needed to clarify the pathogenesis and other possible risk factors for this condition, and to establish if regular screening of kidney ultrasound in these patients can be indicated.

## Introduction

The term congenital adrenal hyperplasia (CAH) refers to a group of inherited autosomal recessive disorders characterized by the impairment of enzymes involved in the adrenal steroidogenesis [1].

21-hydroxylase deficiency (21-OHD) represents the most frequent form of CAH, accounting for more than 90% of cases [2]. Depending on the residual enzymatic activity, 21-OHD shows a wide spectrum of clinical forms, ranging from severe or classical (salt wasting, SW, and simple virilizing, SV) to mild “late onset” or non-classical [3]. Treatment of 21-OHD includes life-long replacement therapy with glucocorticoids and mineralocorticoids. Although steroid replacement therapy allows patients to prevent adrenal crisis, the management of this conditions is complicated by associated comorbidities resulting from both hormonal imbalances and treatment-related effects, such as reduction in growth potential, precocious puberty, and adverse consequences on cardiovascular system and bone [4–9].

In the last twenty years, the incidence of nephrolithiasis in pediatric age has increased from 4 to 10% in children living in industrialized countries [10]. This condition in children substantially differs from adults in several aspects, including the epidemiology, etiology, and symptoms. Microcalculi, defined as ultrasonographic detection of hyperechogenic deposits smaller than 3 mm in diameter, can be detected in renal calyces, pelvis or ureter [11–13], and can represent the first step in stone formation. The composition of stones in children is mostly of oxalate and calcium. Furthermore, in pediatric age the symptoms of nephrolithiasis can be non-specific, and this condition often proceeds asymptomatically.

Genetic and metabolic disorders leading to increased urinary excretion of calcium, oxalates and phosphates, decreased urine volume, urinary supersaturation, insufficient concentrations of crystallization inhibitors, such as citrate and magnesium, represent the most common causes of nephrolithiasis in children [14, 15]. Recently, an increased risk of hypercalcemia, hypercalciuria and nephrocalcinosis has been reported in children with CAH, although the underlying pathogenetic mechanisms are unknown [16, 17].

In this study, we aimed to investigate the incidence of nephrolithiasis in a cohort of children affected with classic form of 21-OHD (SW and SV), and the possible association with the metabolic control of the disease and with glucocorticoid and mineralocorticoid replacement treatment.

## Subjects and methods

This study was designed as a multicenter 1-year prospective study involving five Italian Pediatric Endocrinology Centers. A total of 52 subjects (35 males) with confirmed molecular diagnosis of classic form of 21-OHD (43 SW, 9 SV) were enrolled. Each patient was evaluated at three different times: at the beginning of the study (T0), after 6 months (T1), and 12 months (T2) of follow up. The study was conducted in accordance with the Declaration of Helsinki, and the study protocol was approved by the Local Ethics Committees. An informed consent form was signed by the patients’ parents.

### Clinical data collection

Data about a detailed family history of kidney stones, adrenal crisis episodes, hydrocortisone dose per body surface area, and fludrocortisone dose were collected. Systolic blood pressure (SBP), and diastolic blood pressure (DBP) were evaluated. Body weight and height were measured using standard techniques at T0, T1 and T2. Body mass index (BMI) was calculated using the formula weight/height^2^ (kg/m^2^). Auxological data were expressed as standard deviation score (SDS) for age and gender according to Italian growth charts [18]. Pubertal stage was recorded according to Marshall and Tanner stage [19].

### Laboratory data collection

Blood samples were collected at 08:00 a.m. after an overnight fasting. The serum levels of sodium (Na), potassium (K), calcium (Ca), phosphate (P), creatinine, adrenocorticotrophic hormone (ACTH), 17-hydroxyprogesterone (17-OHP), Δ4-androstenedione, dehydroepiandrosterone sulfate (DHEAS) were measured at baseline, T1 and T2 by using radioimmunoassay technique. Serum active intact parathyroid hormone (PTH) and 25-OH vitamin D were measured by immunological tests based on the principle of chemiluminescence. Ca and P serum concentrations were measured by nephelometric method. Urinary calcium/creatinine (Ca/Cr) ratio, urinary oxalates and citrates excretion were also assessed. Hypercalciuria was defined as Ca/Cr ratio above 0.2.

### Imaging data collection

A renal ultrasonography was performed at each time point of the study by experienced pediatric radiologists by using a convex probe with a frequency between 2.5 and 4 MHz (ultrasound machine Logiq E9, General Electric HealthCare, Chicago, Illinois, USA).

### Statistical analysis

Statistical analyses and data management were performed using SigmaPlot 12.0 for Windows and Chemometric Agile Tool (CAT). All numerical variables were evaluated regarding normality in distribution both graphically (box plots) and statistically using Shapiro-Wilk test, and regarding the homogeneity of variances verified using Levine’s test. Variables normally distributed were expressed as mean ± standard deviation (SD), while variables with non-normal distribution were expressed as median and interquartile range (IQRs).

Statistical comparisons between subjects with and without nephrolithiasis were performed by using Student’s test for normally distributed variables and Mann–Whitney U test for not normally distributed variables.

Statistical comparisons of variables were performed using parametric or non-parametric ANOVA on repeated measures. The level of significance was set in all cases at *p* < 0.05.

A multivariate statistical approach using principal component analysis (PCA) was performed to identify the trend of variables during the time and groupings or the occurrence of regularities and distributions among variables. Two preliminary tests to establish the possibility to run a PCA were performed. The first test was the Kaiser-Meyer-Olkin test (KMO test), a measure of sampling adequacy to evaluate whether the variables considered were consistent for the use of a principal component analysis (its value ranging from 0 to 1 and is consistent above 0.70). The second test was the Bartlett test performed to test the null hypothesis of non-correlation between variables; it provided a statistically significant result with *p* < 0.001. Moreover, before starting PCA, a column autoscaling of data (Z-score values) were performed. Two plots are needed to obtain an interpretation of PCA: loading plot which allows to understand the importance of each original variable in constructing the components and the type of correlation (positive or negative) between all of them; score plot which allows to evaluate the behavior of the data in the new orthogonal space defined by the principal components highlighting similarities and differences among samples. The variables located in the same area of the plot are positively correlated; variables located in opposite quarters are negatively correlated. Then, the Cochran test was performed to evaluate changes over time in the group of patients with NC. The Cochran test is a non-parametric test for analysing randomized complete block designs where the response variable is a dichotomous variable. The Cochran test assumes that there are “c” experimental treatments (time point) and in “r” blocks (patients).

## Results

### Patient population

Among the 52 recruited subjects, 35 (67%) were male and 17 (33%) females. Clinical characteristics and metabolic markers of the entire cohort at baseline and during the follow up are reported in Table 1. All subjects were receiving hydrocortisone at a mean dose of 14.6 ± 5.1 mg/m^2^ that did not change during the study period (*p* > 0.05). Forty-three out of the 52 21-OHD subjects were affected by the SW form and were also receiving fludrocortisone therapy (0.1 mg/day). Eight subjects at T0, 12 subjects at T1 and 11 subjects at T2 were also receiving cholecalciferol supplementation at the dose of 1000 UI per day.

**Table 1:** Comparison of clinical and metabolic markers over time in the whole cohort

Metabolic markers	*T*_*0*_	*T*_*1*_	*T*_*2*_	*p*_*value*_
Chronological age (years)	9.3 ± 4.8	9.7 ± 4.7	10.2 ± 4.7	–
Weight (SDS)	0.8 ± 1.2	0.9 ± 1.1	0.8 ± 1.2	n.s.^b^
Height (SDS)	0.04 ± 1.2	0.05 ± 1.3	0.03 ± 1.3	n.s.^b^
BMI (SDS)	0.9 ± 1.0	1.0 ± 1.1	0.9 ± 1.1	n.s.^b^
SBP (mmHg)	107.0 (105∼120)	114.5 (102.5–120.0)	110.5 (105–120)	n.s.^a^
DBP (mmHg)	67.8 ± 11.8	70.2 ± 13.5	68.0 ± 12.7	n.s.^b^
17-OHP (ng/mL)	11.1 (3.0–25.1)	7.1 (1.8–19.9)	5.9 (2.0–20.0)	*0.005^a^
DHEAS (mcg/dL)	7.2 (1.2–20.7)	3.0 (0.2–16.3)	9.9 (1.9–25.2)	n.s^a^
Δ4-Androstendione (ng/mL)	0.9 (0.3–2.5)	0.3 (0.3–1.1)	0.5 (0.3–1.5)	*0.014^a^
ACTH (pg/mL)	58.5 (16.0–195.5)	57.7 (14.6–187.5)	35.0 (16.0–116.2)	n.s.^a^
Ca/Cr ratio	0.08 (0.05–0.16)	0.11 (0.05–0.17)	0.09 (0.06–0.18)	n.s.^a^
25-OH VitD (ng/mL)	22.0 (17.7–25.0)	24.0 (17.4–28.0)	26.0 (22.7–30.9)	*0.004^a^
PTH (pg/mL)	27.2 (23.0–34.5)	28.0 (24.3–40.9)	25.2 (17.2–38.6)	n.s.^a^
Ca (mg/dl)	9.8 ± 0.5	9.9 ± 0.4	9.6 ± 0.4	n.s.^b^
P (mg/dl)	4.8 ± (4.2–5.2)	4.6 (4.1–5.3)	4.6 (4.2–5.0)	n.s ^b^
Na (mEq/L)	139 (138–140)	139 (138–140)	139 (138–140)	n.s.^b^
K (mEq/L)	4.5 ± 0.5	4.4 ± 0.4	4.4 ± 0.4	n.s.^b^

*17-OHP* 17-hydroxyprogesterone progesterone, *DHEAS* dehydroepiandrosterone sulfate, *ACTH* adrenocorticotrophic hormone, *Ca/Cr* calcium/creatinine ratio

Data are presented as mean ± SD for normally distributed variables, and median with interquartile range for non-normally distributed variables

^a^Non-parametric ANOVA on repeated measures

^b^ANOVA on repeated measures

The superscripts * above to each value indicates statistically significant difference (*p* < 0.05) in the amount of variable over time

### Laboratory data

In the Table 1 are reported the values of each variable at the three time points. A statistically significant difference was found for 17-OHP (*p* = 0.005), with median values ranging from 11.1 (3.0–25.1) ng/mL at T0 to 5.9 (2–20) ng/mL at T2, and for Δ4-androstenedione, with median values ranging from 0.9 (0.3–2.5) ng/mL at T0 to 0.5 (0.3–1.5) ng/mL at T2 (*p* = 0.014). ACTH levels showed a strong downward trend from T0 (median value 58.5 pg/mL) to T2 (median value 35 pg/mL), although a statistically significant difference was not observed. A statistically significant difference was also found for 25-OH vitamin D levels which showed an increase from T0 to T2 (*p* = 0.004), probably due to the cholecalciferol supplementation. PTH, Ca, P, Na and K serum concentrations were in the normal range for all patients and stable during the study time. Mean values of the Ca/Cr ratio were stable during the three time-points.

### Incidence of nephrolithiasis

Of the 52 patients recruited for this study, 11 subjects (10 males) showed at least one ultrasound finding of nephrolithiasis, and only one subject showed a single finding of nephrocalcinosis.

At T0, 9 subjects had nephrolithiasis (17.3%), and 1 showed nephrocalcinosis (1.9%); during the follow up, 7 subjects showed nephrolithiasis (13.5%) at T1, and 6 subjects (11.5%) at T2.

Among the 21-OHD subjects showing nephrolithiasis, the most of them had the SW form of 21-OHD (8 at T0, 5 at T1, and 6 at T2), thus they were receiving both hydrocortisone and fludrocortisone.

Among the 11 21-OHD patients who presented with nephrolithiasis, 4 showed this condition in all the three follow-up visits, 2 subjects at T0 and T1, and 5 subjects had a single finding of nephrolithiasis (3 subjects at T0, and 2 subjects at T1). The child who showed nephrocalcinosis at T0 was found to have nephrolithiasis at T1 and T2.

The Table 2 shows the comparison of ACTH, 17-OHP, DHEAS and Δ4-androstenedione values among the three time points in 21-OHD patients with nephrolithiasis. No statistically significant difference among these metabolic markers was found, even if 17-OHP, DHEAS and Δ4-androstenedione levels showed a tendency towards a reduction from T0 to T2.

**Table 2:** Characteristics of 21-OHD subjects with nephrolithiasis

	*T*_*0*_	*T*_*1*_	*T*_*2*_	*p*_*value*_
Age (yr)	7.8 ± 3.2	8.3 ± 3.2	8.9 ± 3.2	–
Sex (M/F)	8/1	6/1	6/0	–
ACTH (pg/ml)	62.5 (25.0–493.0)	168 (42.4–368)	71.5 (16.5–137.4)	n.s.
17-OHP (ng/ml)	37.5 (12.8–85.3)	19.9 (0.99–30.5)	18.3 (3.0–23.6)	n.s.
DHEAS (mcg/dL)	11.0 (0.5–42.0)	10.3 (1.0–58.9)	8.5 (0.4–51.8)	n.s.
Δ4-Androstendione (ng/mL)	2.1 (0.6–3.2)	0.6 (0.2–2.1)	1.5 (0.4–2.7)	n.s.
Hydrocortisone (mg/m^2^)	14.01 ± 4.8	14.8 ± 4.67	15.63 ± 4.67	n.s.

*17-OHP* 17-OH progesterone, *DHEAS* dehydroepiandrosterone sulfate, *ACTH* adrenocorticotrophic hormone, *Ca/Cr*

calcium/creatinine ratio

Data are presented as mean ± SD for normally distributed variables, and median with interquartile range for non-normally distributed variables

Test were performed with non-parametric ANOVA on repeated measures

Although the mean dose of hydrocortisone remained stable in the entire cohort during the study period, the subjects with nephrolithiasis showed an increase in the mean dose even if not statistically significant.

Among the subjects with nephrolithiasis, none of them had a family history of kidney stones; 3 subjects, including the one with nephrocalcinosis, had mild hypercalciuria at T0, 2 subjects at T1 and 2 subjects at T2. During the follow-up period, serum Na, K, Ca, P, PTH levels were in the normal range for all subjects, as well as urinary excretion of Na and K. Urinary oxalate excretion was slightly increased in 2 subjects at T0, while urinary citrate excretion was reduced in 4 subjects at T0 and in 2 subjects at T1 and T2.

Table 3 shows comparisons between subjects with and without nephrolithiasis during the three time points.

**Table 3:** Comparisons of subjects with and without nephrolithiasis over time

	T_0_			T_1_			T_2_		
	Subjects without nephrolithiasis	Subjects with nephrolithiasis	*p*	Subjects without nephrolithiasis	Subjects with nephrolithiasis	*p*	Subjects without nephrolithiasis	Subjects with nephrolithiasis	*p*
Age (yr)	9.6 ± 5.1	8.1 ± 3.4	n.s^a^	9.5 ± 4.7	9.8 ± 4.9	n.s^a^	10.6 ± 4.8	7.1 ± 2.6	n.s^a^
ACTH (pg/ml)	43.2 (10.8–200.3)	62.5 (32.1–320.5)	n.s^b^	54.7 (14.0–167)	60.7 (13.9–167)	n.s^b^	35.0 (16.0–114.4)	123.0 (14.9–595.5)	n.s^b^
17-OHP (ng/ml)	8.2 (2.3–18.6)	37.5 (14.0–104.5)	0.02^b^	7.7(2.2–20)	6.9 (2.3–19.5)	n.s^b^	5.0 (1.9–20.0)	23.6 (3.6–99.6)	n.s^b^
DHEAS (mcg/dl)	7.1 (1.8–16.1)	11.0 (1.1–43.8)	n.s^b^	3.4(0.4–42.4)	3.2 (0.3–34.3)	n.s^b^	14.5 (1.9–37.4)	8.5 (0.4–42.4)	n.s^b^
Δ4-Androstenedione (ng/ml)	0.8 (0.3–2.4)	2.1 (0.5–3.4)	n.s^b^	0.9 (0.5–2.1)	0.96 (0.7–2.4)	n.s^b^	0.9 (0.3–2.1)	0.9 (0.3–2.5)	n.s^b^
Hydrocortisone (mg/m^2^)	15 ± 5.2	13.2 ± 4.8	n.s^a^	14.2 ± 4.8	12.2 ± 4.8	n.s^a^	14.6 ± 4.4	14.9 ± 6.3	n.s^a^

*17-OHP* 17-hydroxyprogesterone progesterone, *DHEAS* dehydroepiandrosterone sulfate, *ACTH* adrenocorticotrophic hormone, *n.s* non significant

Data are presented as mean ± SD for normally distributed variables, and median with interquartile range for non-normally distributed variables

^a^Comparisons performed with Student’s test

^b^Comparisons were performed with Mann–Whitney U test

### PCA of metabolic markers

**Fig. 1:**
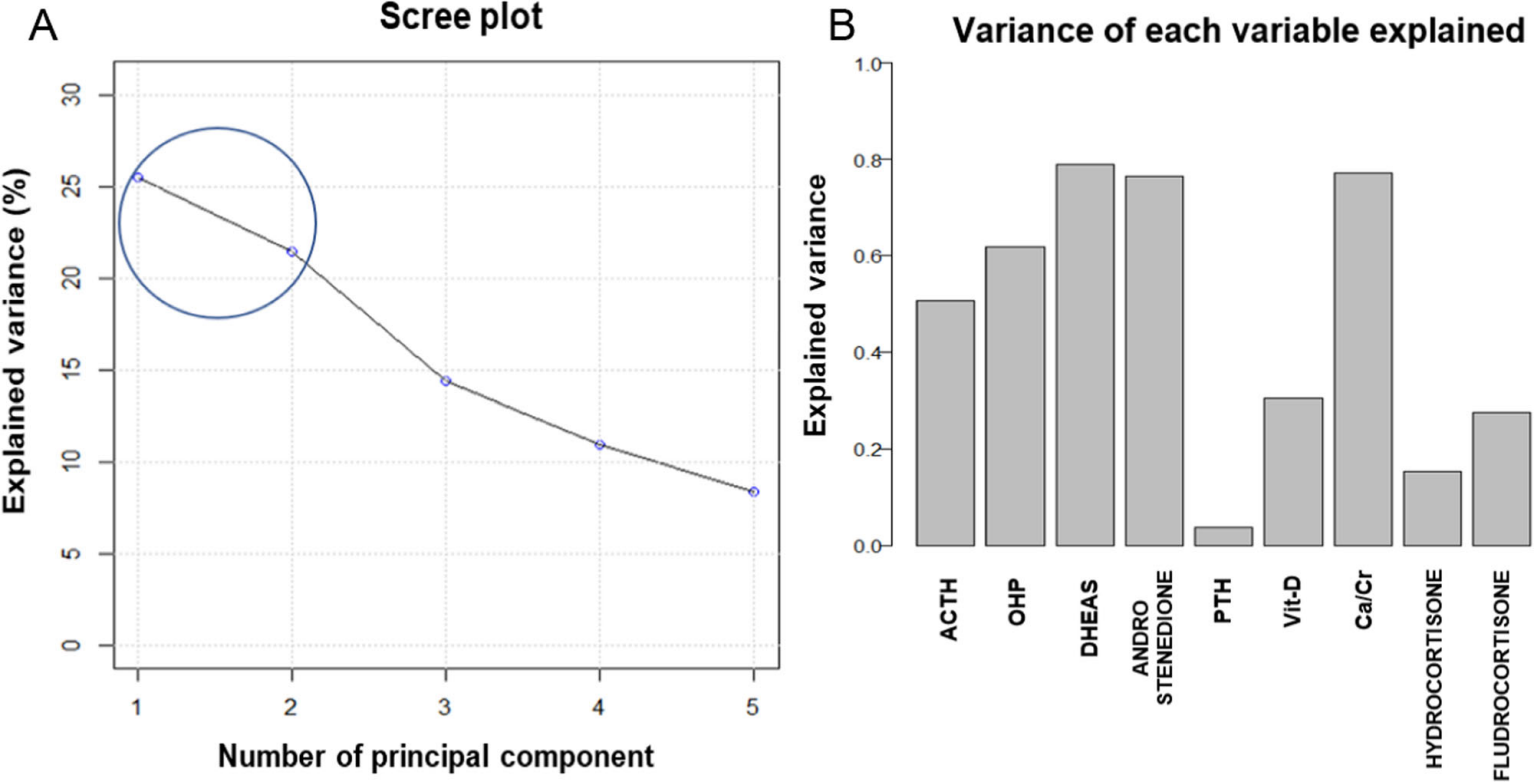
**A**, **B** Scree plot: percentage of explained variance vs number of principal component of the model (in general the components number before the inflection point are retained); variance of each variable explained: the weight of each variable to the two principal components selected in the PCA model

**Fig. 2:**
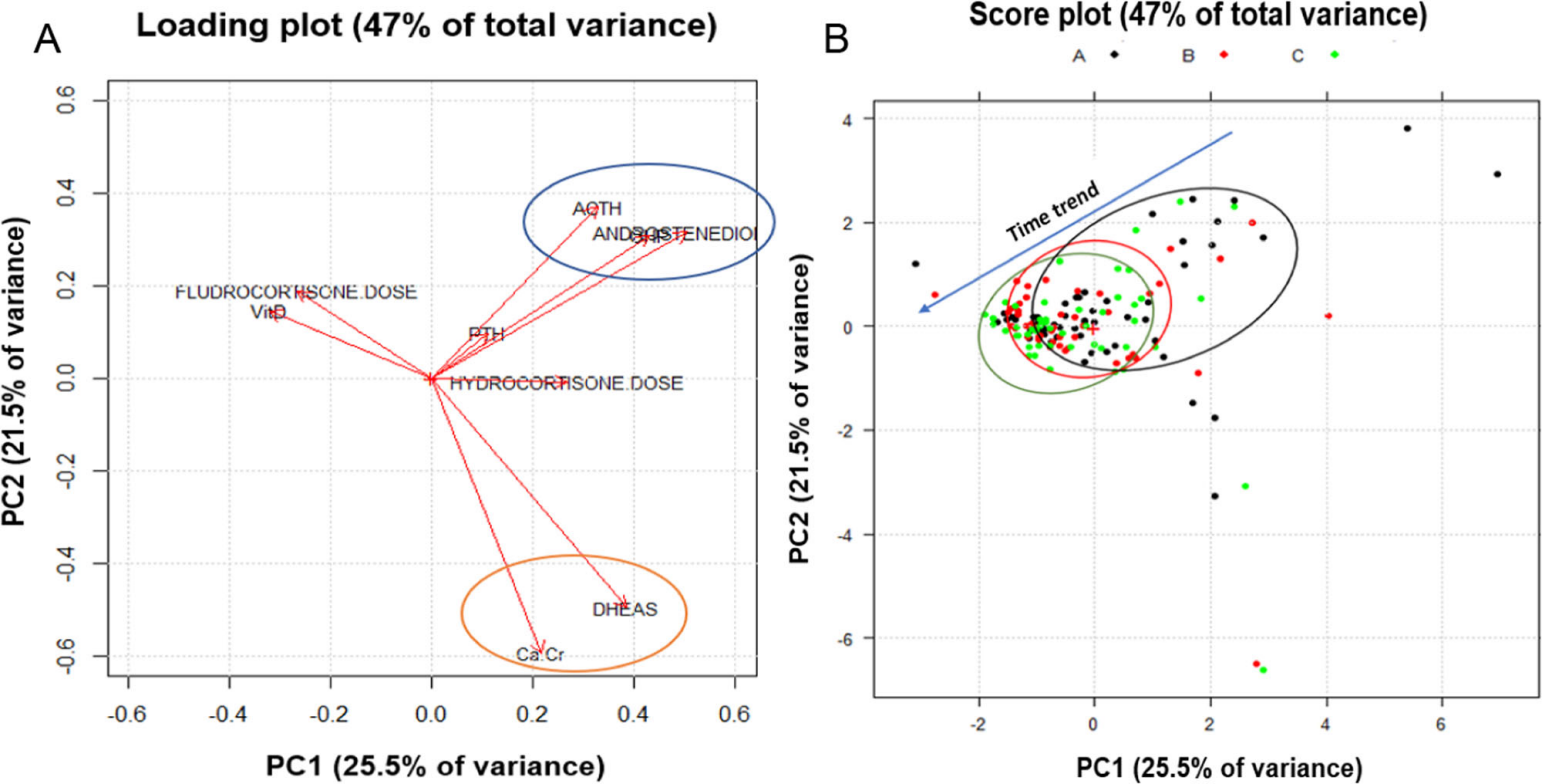
**A**, **B** Loading and score plots of PCA (principal components 1 and 2) of variables. In the loading plot, blue and orange circles represent correlation patterns between variables. In the score plot, black, red and green circles (and respective dots **A**–**C**) represent groupings of the variables amount at starting time point, first time point, and the end point, respectively

Two principal components were fixed as observed by scree plot (Fig. 1A). The first and the second principal component (PC1 and PC2) explained 25.5 and 21.5% of overall variance, respectively, with a total variance explained equal to 47%. The importance of each variable to build principal components was investigated. Interestingly, as shown in Fig. 1B, DHEAS, Ca/Cr ratio, 17-OHP, Δ4-androstenedione variables explained, each one, more than 60% of variance, followed by ACTH (50% of variance explained), and the others that explained not much about variability over time. These results were confirmed observing loading plot in Fig. 2A: variables with longer vectors had a strong weight in the principal components, while markers with short vector did not have relevance in explaining variability and consequently differences over time. Moreover, from loading plot, correlations between variables were observed. In particular, two strong correlation patterns among 17-OHP, Δ4-androstenedione and ACTH, and among DHEAS and Ca/Cr ratio were found (Fig. 2A, blue and orange circles). Considering the score plot (Fig. 2B), a trend over time points (black, red and green circles) along negative direction of PC1 and PC2 was found. As the trend was observed along the negative directions of PC1 and PC2, moving from the first time point (black dot) to the end point (green dot), these results suggested that a decrease in the 17-OHP, Δ4-androstenedione and ACTH amounts occurred during the follow up.

The decrease in the variables like 17-OHP, Δ4-androstenedione and ACTH over time, matched with the decrease in the cases of nephrolithiasis during the follow up.

## Discussion

In this study we evaluated the incidence of nephrolithiasis in a cohort of 52 children affected with 21-OHD during a 1 year of follow up. At the beginning of the study, we found a percentage of nephrolithiasis of 17.3% which decreased to 11.5% at the end of the study, although this reduction was not statistically significant. It is important to note that the observed incidence of nephrolithiasis in our cohort is significantly higher than that reported in the pediatric population [10]. Moreover, the incidence of nephrolithiasis was highest at the beginning of the study when the subjects showed worse metabolic control. The prevalence of nephrocalcinosis instead was 1.9% similar to that reported in the general pediatric population [20, 21]. Hypercalciuria is considered one of the most important risk factors for nephrolithiasis [22]. It can result from idiopathic hypercalciuria, a multifactorial disease, as well as from genetic disorders or other underlying diseases, such as (primary) hyperparathyroidism. Anatomical abnormalities of the kidney, urinary tract infections, insufficient fluid uptake, and obesity are known to predispose to nephrolithiasis [23].

In our cohort, among the 12 subjects with ultrasound determination of nephrolithiasis and nephrocalcinosis, hypercalciuria was identified in 7 cases. Oxalates urinary excretion was observed only in 2 subjects at T0 and in no one at T1 and T2. Hyperoxaluria is a condition due to increased endogenous production of oxalate in the liver (primary hyperoxaluria), or increased intestinal absorption of oxalate or increased dietary intake of oxalate and its metabolic precursors (secondary or enteric hyperoxaluria). Citrate is an important inhibitor of stone formation in the urine as it prevents the binding of calcium with other ions such as oxalate and phosphate, and increases the solubility of calcium oxalate by increasing the pH of urine. Thus, hypocitraturia is a significant risk factor for nephrolithiasis. In our cohort hypocitraturia was observed in 4 subjects at T0 and in 2 subjects at T1 and T2.

To date, the pathogenetic mechanisms linking nephrolithiasis with 21-OHD are unknown. Schoelwer et al., examined retrospectively the prevalence of hypercalcemia, hypercalciuria and nephrocalcinosis in a cohort of 40 subjects with classic form of CAH. The authors found that 33 subjects (84%) had hypercalcemia, 6 (15%) hypercalciuria, and 3 (6%) nephrocalcinosis. Interestingly, a positive correlation between calcium serum levels and 17-OHP was found [16].

Madihi et al., investigated the prevalence of nephrolithiasis in a cohort of 120 iranian children affected

with CAH, and they found nephrolithiasis in 4 male subjects (3.3%) [17].

Aswani et al., reported the finding of renal cysts and nephrocalcinosis in a 11 years-old children affected by 11ß-hydroxylase deficiency, a less common form of CAH characterized by hypertension and symptoms of androgens excess [24]. The patient did not show any of the common causes of nephrocalcinosis, and the authors associated the renal alterations to the chronic interstitial nephritis due to the longstanding hypokalemia [25].

In our study, we found that the whole cohort of 21-OHD patients showed a statistically significant trend towards the reduction in the serum levels of ACTH, 17-OHP and Δ4-androstenedione, which are important indicators of the metabolic control of the disease, during the study period. A similar trend for 17-OHP, Δ4-androstenedione and DHEA-S was found in the subjects with nephrolithiasis, even if not statistically significant. In parallel with the improvement of metabolic control, probably due to the increased dose of hydrocortisone, we found a relevant reduction, even if not statistically significative, of the cases of subjects affected with nephrolithiasis. Indeed, at the end of the study three subjects no longer showed nephrolithiasis suggesting that accurate control of the disease may prevent the development of this condition.

## Conclusions

To our knowledge this is the first prospective study investigating the prevalence of nephrolithiasis in a cohort of children with 21-OHD. We determined the incidence of this condition, and for the first time we established an association between nephrolithiasis and the metabolic control of the disease.

Limitations of the study are the assessment of the biochemical parameters with different laboratory kits in different centers, and the execution of renal ultrasound by different pediatric radiologists.

Additional studies are needed to clarify the pathogenesis and other possible risk factors of nephrolithiasis in children with 21-OHD, and to establish if regular screening by renal ultrasound in these patients can be indicated.
